# A single preoperative low-dose dexamethasone may reduce the incidence and severity of postoperative delirium in the geriatric intertrochanteric fracture patients with internal fixation surgery: an exploratory analysis of a randomized, placebo-controlled trial

**DOI:** 10.1186/s13018-023-03930-2

**Published:** 2023-06-19

**Authors:** Jian-wen Huang, Yun-fa Yang, Xiao-sheng Gao, Zhong-he Xu

**Affiliations:** Department of Orthopaedic Surgery, Guangzhou First People’s Hospital, The Second Affiliated Hospital, School of Medicine, South China University of Technology, 1 Panfu Road, Guangzhou, 510180 Guangdong China

**Keywords:** Postoperative delirium, Dexamethasone, Intertrochanteric fractures, The elderly

## Abstract

**Objective:**

Postoperative delirium (POD) is a common complication along with poor prognosis in geriatric intertrochanteric fracture (ITF) patients. However, the prevention and treatment of POD remain unclear. Previous studies have confirmed that POD is essentially a consequence of neuro-inflammatory responses. Dexamethasone is a glucocorticoid with comprehensive anti-inflammatory effects, while a high dose of dexamethasone correlates with many side effects or even adverse consequences. Thus, this prospective study aims to discuss whether a single preoperative low-dose dexamethasone can reduce the impact of POD on geriatric ITF patients with internal fixation surgery.

**Methods:**

Between June 2020 and October 2022, there were 219 consecutive ITF patients assessed in our department. Of the 219 ITF patients, 160 cases who met the inclusion and exclusion criteria were finally enrolled and randomly allocated to the dexamethasone group and the placebo group (80 geriatric ITF patients in each group) in this prospective study. The patients in the dexamethasone group received intravenous 10 mg (2 ml) dexamethasone while the patients in the placebo group received intravenous 2 ml saline in 30 min before being sent to the operating room, respectively. The baseline characteristics, surgical information, incidence and severity of POD as the efficacy-related outcomes, and infection events and hyperglycemia as safety-related outcomes (adverse events), were collected and analyzed between the two groups. The severity of POD was evaluated by Memorial Delirium Assessment Scale (MDAS) score.

**Results:**

There were no differences in baseline characteristics and surgical information between the dexamethasone group and the placebo group. The dexamethasone group had a lower incidence of POD than the placebo group within the first 5 days after surgery [(9/80, 11.3% vs. 21/80, 26.3%, RR = 0.83, 95% CI 0.71–0.97, *P* = 0.015]. The dexamethasone group had lower MDAS scores (Mean ± SD) than the placebo group [13.2 ± 1.0 (range 11 to 15) vs. 15.48 ± 2.9 (range 9 to 20), *P* = 0.011, effect size = 0.514]. There were no differences in infection events and hyperglycemia between the two groups.

**Conclusions:**

A single preoperative low-dose dexamethasone may reduce the incidence and severity of POD in geriatric ITF patients with internal fixation surgery.

*Trial registration*: ChiCTR2200055281.

**Supplementary Information:**

The online version contains supplementary material available at 10.1186/s13018-023-03930-2.

## Introduction

Intertrochanteric fracture (ITF), also commonly known as one kind of hip fracture, is important causes of hospitalization in the elderly. The population aged over 70 years will double in the next three decades, leading to a great number of ITF globally [1, 2]. ITF is typically treated with surgery because conservative treatment is associated with high mortality and poor functional recovery [3]. However, postoperative delirium (POD), defined as an acute disruption in attention, consciousness, and cognition, is a common and serious complication following ITF surgery, with reported incidences varying from 14.3 to 25.0% [4–6]. Patients who develop POD are associated with prolonged hospital stays, high medical costs, postoperative cognitive impairment, and increased mortality [7].

Despite the high prevalence and morbidity of POD, the exact pathophysiology is still not fully elucidated. Various mechanisms are proposed to understand POD, of which the most widely recognized one is the neuro-inflammatory mechanism [8, 9]. Concerning ITF, the first trauma, subsequent anesthesia, and following surgery trigger both local and systemic inflammatory responses, which lead to an increase of pro-inflammatory and anti-inflammatory mediators. As a result, this can cause neuronal dysfunction or synaptic impairment, and finally, delirium [10, 11].

Corticosteroids, which are routinely used for the treatment of postoperative complications, are potent suppressors of inflammatory response. The effects of corticosteroids on reducing various inflammatory factors have been confirmed in those patients who underwent surgery [12–14]. Therefore, many researchers try to prevent or reduce POD with corticosteroids. Previous studies have demonstrated that preoperative corticosteroids could reduce the risk of POD in hip fractures [15–17]. In a double-blind, randomized, placebo-controlled feasible trial, Kluger et al. revealed that a single dose of 20 mg intravenous dexamethasone at hospital admission was associated with a reduced POD severity but not the occurrence in hip fracture patients [17]. However, it is not clear the safety of dexamethasone for patients with acute fractures in such a high dose. Moreover, the timing of dexamethasone used at hospital admission is probably too early for anesthesia and surgery. If the drug efficacy of the hospital admission dose of dexamethasone is gone, supplementary uses are prudent due to increasing the complication risks like infection and hyperglycemia. Hereby, we think that a low-dose dexamethasone intravenously used within 30 min before being sent to the operating room is not only safe for aging patients but also effective to reduce the impact of POD.

Therefore, we hypothesize that a single low dose of 10 mg dexamethasone intravenously administrated within 30 min before being sent to the operating room can reduce the incidence and severity of POD in geriatric ITF patients after internal fixation surgery.

## Patients and methods

### Study design

This study was a single-center, single-blinded, randomized, placebo-controlled trial, which complied with the Declaration of Helsinki and received ethics approvals from the ethics committees of the Guangzhou First People’s Hospital (Number: K-2020-106-01). All patients provided written informed consent. The study was registered at the Chinese Clinical Trials Registry (Registration number: ChiCTR2200055281). Patients were recruited by orthopedists between June 2020 and October 2022.

### Participants

All patients were diagnosed with ITF by X-ray. Inclusion criteria: (1) age over 60 years, (2) required surgical treatment, (3) fracture within three weeks, and (4) capable of complying with the study protocol. Exclusion criteria: (1) systemic or fracture site infection before, (2) multiple traumas or open fractures, (3) stroke within 3 months or acute myocardial infarction within 6 months, (4) active peptic ulcer, (5) pathological fracture or pending fracture diagnosed by radiographic data, (6) chronic organic failure without effective replacement therapy, (7) advanced malignant tumor, (8) severe cognitive disorder (Mini-mental State Examination, MMSE ≤ 9 points) [18], (9) severe malnutrition (body mass index, BMI < 16) or overnutrition (BMI ≥ 35), (10) medication of systemic corticosteroids more than two weeks, and (11) patients who are considered to be inappropriate for participation by researchers.

### Randomization

After the initial screening, all the patients were randomized into two groups (the dexamethasone group *vs.* the placebo group) with an allocation ratio of 1:1. The random number generated via SPSS (IBM SPSS Statistic for Windows, Version 25.0. Armonk, NY: IBM Corp) by a clinical research coordinator who was not involved in the study, and the results were concealed from the researchers. The assignment list was sealed in opaque envelopes, and they were opened to determine dexamethasone or placebo by the researchers after obtaining informed consent.

### Baseline variables

Baseline characteristics included gender, age, Body Mass Index (BMI), fracture site, American Society of Anesthesiologists (ASA) classification, comorbidities, pre-injury living condition, Activities of Daily Life (ADL) [19], nutrition status according to Mini-Nutritional Assessment (MNA) [20], preoperative cognitive level according to Mini-Mental State Examination (MMSE) [18], hemoglobin and albumin levels at admission were collected. In addition, surgical data including fixation type, the timing of surgery, surgical time, anesthesia, blood loss, and intraoperative transfusion were collected.

### Interventions

All ITF patients received treatments and interventions in the setting of standard Enhanced Recovery After Surgery (ERAS) procedure, of which the management content mainly included three parts included preoperative, intraoperative, and postoperative management. The preoperative management included education programs, auxiliary examination nutrition, nutrition management, pain management, DVT prevention, sleep management, and gastrointestinal management. On the morning of surgery, those patients allocated to the dexamethasone group received intravenous 10 mg (2 ml) dexamethasone (Dexamethasone Sodium Phosphate Injection, Tianjin Pharmacy), while the patients allocated to the placebo group received intravenous 2 ml physiological saline (0.9% sodium chloride injection) in 30 min before being sent to the operating room. The dexamethasone or physiological saline was given by an independent nurse who was not involved in the trial and was blinded to the patient’s outcome. All patients in this study underwent standard anesthesia procedures (including receiving routine sedatives and care) determined by the anesthesiologists who were blinded to patient allocation. Standard endotracheal general anesthesia was induced sequentially with sufentanil (0.5–0.7 μg/kg), cis-atracurium (0.16–0.2 mg/kg), and propofol (2–2.5 mg/kg) was maintained with propofol and sevoflurane. Remifentanil (0.05–2 μg/kg/min) was given intraoperatively as needed at the discretion of the anesthesiologists. Spinal anesthesia was administered with ropivacaine 0.5% (1.5–2.5 ml). Ultrasound-guided psoas compartment block was administered with ropivacaine 0.5% (20–30 ml). The intraoperative part included anesthesia selection, surgery optimization, and temperature management. The postoperative part included pain management, DVT prevention, respiratory management, and functional exercise. A detailed description of the ERAS procedure can be found in Additional file 1. POD was managed according to routine clinical practice. Nonpharmacological measures (such as visual/hearing aids, noise reduction, and avoidance of unnecessary indwelling catheters) were first performed. If the symptoms worsen or do not resolve, pharmacological measures would be considered according to the specialty consultant (0.25–1 mg of oral/intravenous haloperidol is given every 12 h as needed until symptom resolution).

### Outcomes measures

The efficacy-related outcomes reported in this study were the incidence and severity of POD in the first 5 days after surgery. Patients were screened for POD using the Nursing Delirium Screening Scale (Nu-DESC) [21], an easy-to-use tool for assessing delirium in routine nursing care without requiring specialized knowledge [15]. The first screening was performed within 8 h after internal fixation surgery, and routine screening was performed twice a day (at 8 h intervals) in the first 5 days after surgery. A score of 2 or above on two consecutive assessments suggested possible POD. Afterward, patients suspected of POD were evaluated using the Memorial Delirium Assessment Scale (MDAS) by one researcher who was blinded to assignment twice a day [22]. It consisted of 10 items with a maximum score of 30, which assessed cognitive function including memory, attention, orientation, psychomotor activity, and so on. POD severity was determined by the average scores of MDAS.

Safety-related outcomes were infection events (including wound infection, postoperative pneumonia, and urinary infection), hyperglycemia, and maximum glucose in the first 3 days postoperatively (Additional file 1).

### Sample size

The pre-specified sample size was calculated according to the primary outcome (1-year mortality after surgery) of a randomized, placebo-controlled trial. The incidence of primary outcome was as high as 23% or even 36% in previous studies [23, 24]. We hypothesized that 1-year mortality after surgery in the placebo group and the dexamethasone group was 36% and 15%, respectively. Therefore, we calculated that a total of 160 patients (80 patients in each group) were enough to detect the difference with a 5% type I error and a power of 80% in a two-sided test. The recruitment time was expected to be 16 months (from June 2020 to October 2021). However, affected by the COVID-19, the recruitment and study were extended to Oct 2022.

### Statistical analysis

All analyses of related outcomes were performed according to the intention-to-treat principle. Continuous variables were assessed for normal distribution with a Q–Q plot and Kolmogorov–Smirnov test. Students *t* test were applied for normally distributed continuous variables and the Mann–Whitney *U*-test for non-normally distributed continuous variables. Chi-squared or Fisher’s exact tests were used to assess differences for categorical variables. Effect sizes for outcome variables were estimated according to the superiority effect size measure, of which the difference ≥ 0.56, ≥ 0.64, and ≥ 0.71 are considered small, medium, and large, respectively [25]. All statistical analyses were performed with SPSS Version 25.0. A *p* value below 0.05 was considered statistically significant.

Subgroup analyses were done post hoc to explore whether the overall primary outcome differed between groups (including gender, age, ASA, nutrition, cognitive levels, anemia, pain degree, time of surgery, surgery time, and anesthesia). The results are presented as a forest plot with the RRs and 95% CIs as well as *p* for interaction. The forest plot was generated using the “forestplot” *R* package in *R studio* (Version 4.1.2).

## Results

**Fig. 1:**
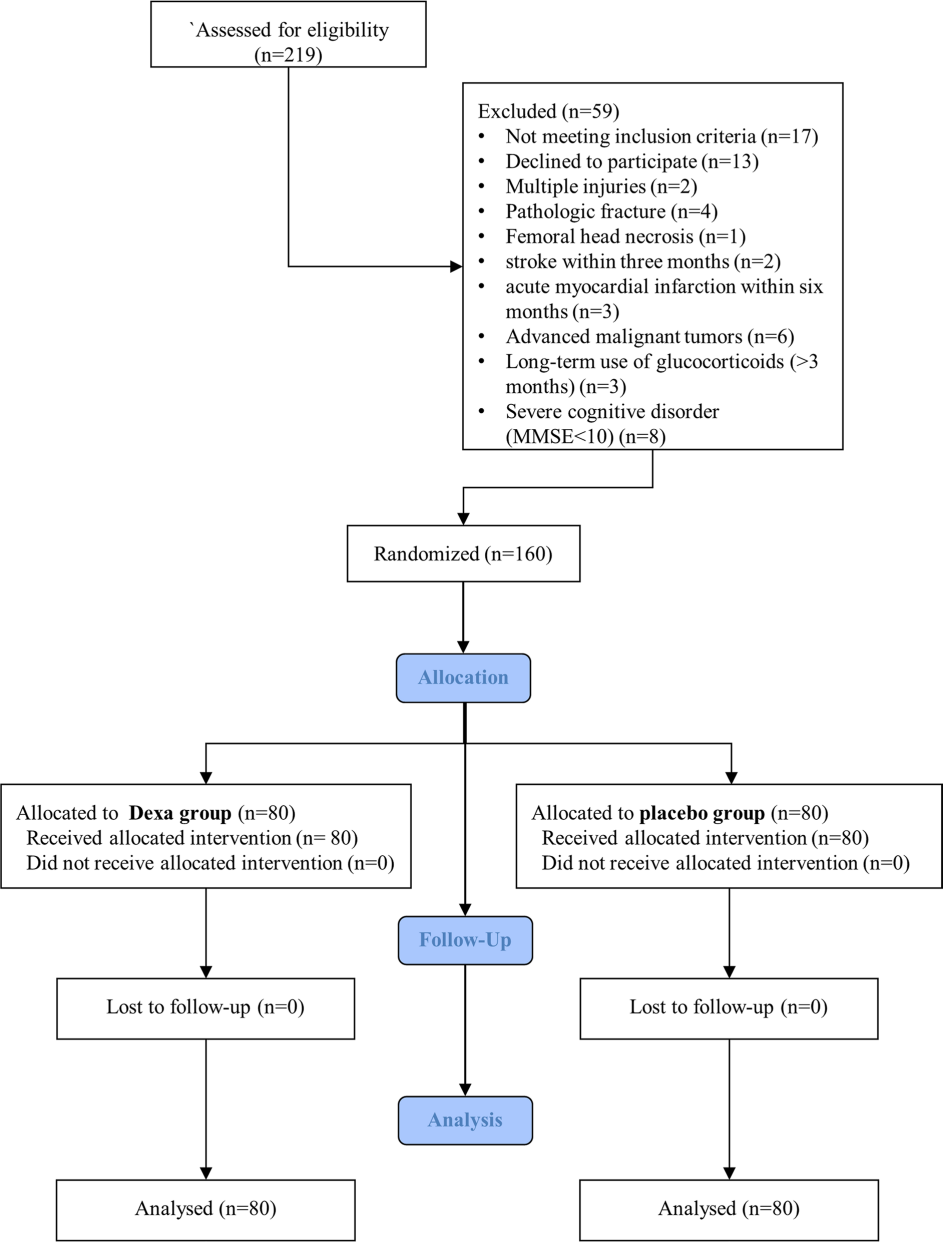
Flow diagram

Between June 2020 and October 2022, a total of 219 patients were assessed for eligibility. Seventeen patients (7.76%) who did not meet the inclusion criteria and 42 patients (19.18%) who fulfilled the exclusion criteria were excluded. Finally, 160 patients were randomly assigned to either the dexamethasone group or the placebo group. No patient was withdrawn from the study. The flow chart is presented in Fig. 1

Baseline characteristics and surgical data between groups did not have statistical differences (Tables 1, 2). Median (IQR) time from admission to intervention (nearly coincide with the time of surgery) in dexamethasone and placebo groups were approximately 89.9 [57.6, 135.6] and 94.3 [66.0, 142.5] hours; the difference was not significant (*p* = 0.228) (Table 2).

**Table 1:** Demographic characteristics of all patients (values are presented as number (%) or median [IQR])

	Placebo (*n* = 80)	Dexa (*n* = 80)	*P*
Male (%)	32 (40.0)	30 (37.5)	0.871
Age, years	85.0 [79.8, 90.2]	84.5 [79.0, 89.0]	0.514
BMI	20.3 [19.2, 21.5]	20.1 [19.3, 20.8]	0.342
ASA ≥ 3 (%)	66 (82.5)	68 (85.0)	0.83
Pain degree at admission (%)			0.45
Mild	6 (7.5)	4 (5.0)	
Moderate	60 (75.0)	56 (70.0)	
Severe	14 (17.5)	20 (25.0)	
*Comorbidities*			
Hypertension (%)	61 (76.2)	58 (72.5)	0.717
CAP or COPD (%)	38 (47.5)	33 (41.2)	0.524
COPD (%)	13 (16.3)	8 (10.0)	0.242
Diabetes (%)	26 (32.5)	22 (27.5)	0.605
CHF or CHD (%)	31 (38.8)	27 (33.8)	0.622
Antiplatelet therapy (%)	18 (22.5)	17 (21.2)	1
Malignant tumor (%)	4 (5.0)	4 (5.0)	1
Chronic kidney disease (%)	10 (12.5)	9 (11.2)	1
*Pre-injury mobility and status*			
ADL			0.09
21–40	21 (26.3)	10 (12.5)	
41–59	28 (35.0)	33 (41.3)	
61–100	31 (38.7)	37 (46.2)	
MNA	10.0 [8.0, 11.2]	10.0 [8.8, 11.0]	0.591
MMSE	16.0 [14.0, 23.0]	17.5 [12.8, 22.2]	0.678
Cognitive disorder (%)			0.21
Moderate	53 (66.2)	48 (60.0)	
Mild	15 (18.8)	24 (30.0)	
No	12 (15.0)	8 (10.0)	
Pre-injury living condition (%)			0.296
Independently at home	15 (18.8)	10 (12.5)	
At home with help needed	45 (56.2)	42 (52.5)	
Aged care facility	20 (25.0)	28 (35.0)	

*BMI* body mass index, *ASA* American society of anesthesiologists, *CAP* community acquired pneumonia, *COPD* chronic obstructive pulmonary disease, *CHF* chronic heart failure, *CHD* coronary heart disease, *CKD* chronic kidney disease, *ADL* activities of daily life, *MNA* mini-nutritional assessment

**Table 2:** Blood test at admission and surgical data (values are presented as mean ± SD, number (%) or median (IQR))

	Placebo (n = 80)	Dexa (n = 80)	*P*
WBC, 10^9^/L	10.1 [8.0, 12.1]	9.5 [7.9, 12.3]	0.539
WBC > 12*10^9^/L (%)	21 (26.2)	22 (27.5)	1
NLR	6.7 [4.4, 10.1]	5.8 [4.4, 8.3]	0.308
RBC, 10^12/L	3.6 ± 0.7	3.6 ± 0.6	0.767
Hgb, g/L	104.1 ± 22.4	105.0 ± 17.0	0.778
Hgb < 80 g/L (%)	11 (13.8)	5 (6.2)	0.188
PLT, 10^9^/L	207.0 [165.2, 245.5]	197.5 [168.2, 242.0]	0.672
ALB, g/L	33.2 ± 3.9	33.6 ± 3.5	0.54
ALB < 35 (%)	53 (66.2)	54 (67.5)	1
Hypokalemia (%)	14 (17.5)	16 (20.0)	0.839
Hyperkalemia (%)	5 (6.2)	3 (3.8)	0.717
Hyponatremia (%)	14 (17.5)	12 (15.0)	0.83
Hypernatremia (%)	2 (2.5)	7 (8.8)	0.17
Hypocalcemia (%)	13 (16.2)	11 (13.8)	0.825
Fracture type (%)			0.75
Stable fracture	46 (57.5)	43 (53.8)	
Unstable fracture	34 (42.5)	37 (46.2)	
Fixation (%)			0.275
CMN with blade	52 (65.0)	45 (56.2)	
CMN with screw	27 (33.8)	31 (38.8)	
PFBN	1 (1.2)	4 (5.0)	
Timing of surgery, h	94.3 [66.0, 142.5]	89.9 [57.6, 135.6]	0.228
Surgery time, min	74.0 [60.0, 93.5]	76.0 [62.8, 90.5]	0.484
Anesthesia (%)			0.871
General	32 (40.0)	30 (37.5)	
Spinal	48 (60.0)	50 (62.5)	
Nerve block (%)	37 (46.2)	45 (56.2)	0.268
Blood loss, ml	100.0 [97.5, 200.0]	150.0 [100.0, 250.0]	0.198
Blood transfusion (%)	24 (30.0)	21 (26.2)	0.725
Plan ICU	5 (6.2)	0.263^P^	0.263
No Plan ICU	2 (2.5)	0.440^Y^	0.440

A* p*-value below 0.05 was considered statistically significant

*WBC* white blood cell, *NLR* neutrophil/lymphocyte ratio, *RBC* red blood cell, *Hgb* hemoglobin, *PLT* platelet, *ALB* albumin, *CMN* cephalomedullary nail, *PFBN* proximal femur bionic nail

The efficacy-related outcomes and safety-related outcomes are shown in Table 3. The dexamethasone group had a significantly lower accumulated incidence of POD within the first 5 days after surgery compared with the placebo group (11.3%, 9/80 vs. 26.3%, 21/80, RR = 0.83, 95% CI 0.71–0.97, *P* = 0.015). Moreover, significantly lower MDAS scores (Mean ± SD, range) was also found in the dexamethasone group (13.2 ± 1.0, 11–15) compared with the placebo group (15.48 ± 2.9, 9–20), (*P* = 0.011, effect size = 0.514). Besides, there is a lower accumulated incidence rate of POD on the fourth day after surgery in the dexamethasone group (11.3%, 9/80, vs. 23.8, 19/80, RR = 0.86, 95% CI 0.74–0.99, *P* = 0.037) (Table 4). No significant differences were observed in the safety-related outcomes including wound infections, postoperative pneumonia, urinary infections, hyperglycemia, and maximum glucose. The adverse events were shown in Additional file 1.

**Table 3:** The efficacy-related and safety-related outcomes between the two groups (values are presented as number (%) or median (IQR))

	Placebo (n = 80)	Dexa (n = 80)	Effect size	Relative risk (95 CI%)	*P*
*Postoperative delirium*					
Overall accumulated incidence (%)	21 (26.3)	9 (11.3)		0.83 (0.71–0.97)	**0.015**
Overall MDAS	15.8 ± 2.9 (9–20)	13.2 ± 1.0 (11–15)	0.514		**0.011**
*Safety-related outcomes*					
Pulmonary infections (%)	28 (35.0)	23 (28.7)		0.91 (0.74–1.13)	0.396
Wound infection (%)	1 (1.2)	1 (1.2)		1.00 (0.97–1.04)	1
Urinary infection (%)	4 (5.0)	3 (3.8)		0.99 (0.92–1.06)	1
Sepsis (%)	2 (2.5)	0 (0.0)		NA	0.477
Hyperglycemia (%)	13 (16.2)	21 (26.2)		1.14 (0.97–1.34)	0.176
Maximum glucose (mmol/L)	7.9 [6.5, 12.8]	9.5 [7.3, 12.4]	0.443		0.214

*P* ≤ 0.05, the statistically significant difference shown in bold

**Table 4:** Comparisons of the incidence and accumulated incidence between the groups on the first five days after surgery

	Comparison of POD incidence				Comparison of POD accumulated incidence			
	Placebo	Dexa	P	RR 95% CI	Placebo	Dexa	P	RR 95 CI
Day 0	1 (1.3)	0 (0)	1.000	0.99 (0.96–1.01)	1 (1.3)	0 (0)	1.000	0.99 (0.96–1.01)
Day 1	6 (7.5)	7 (8.8)	0.772	1.01 (0.92–1.11)	7 (8.8)	7 (8.8)	1.000	1.00 (0.91–1.10)
Day 2	5 (6.3)	1 (1.3)	0.212	0.95 (0.89–1.01)	12 (15.0)	8 (10.0)	0.339	0.94 (0.84–1.06)
Day 3	5 (6.3)	0 (0)	0.069	0.94 (0.89–0.99)	17 (21.3)	8 (10.0)	0.05	0.88 (0.76–1.00)
Day 4	2 (2.5)	1 (1.3)	0.084	0.87 (0.75–1.02)	19 (23.8)	9 (11.3)	**0.037**	0.86 (0.74–0.99)
Day 5	2 (2.5)	0 (0)	1.000	0.99 (0.95–1.03)	21 (26.3)	9 (11.3)	**0.015**	0.83 (0.71–0.97)

*P* ≤ 0.05, the statistically significant difference shown in bold

**Fig. 2:**
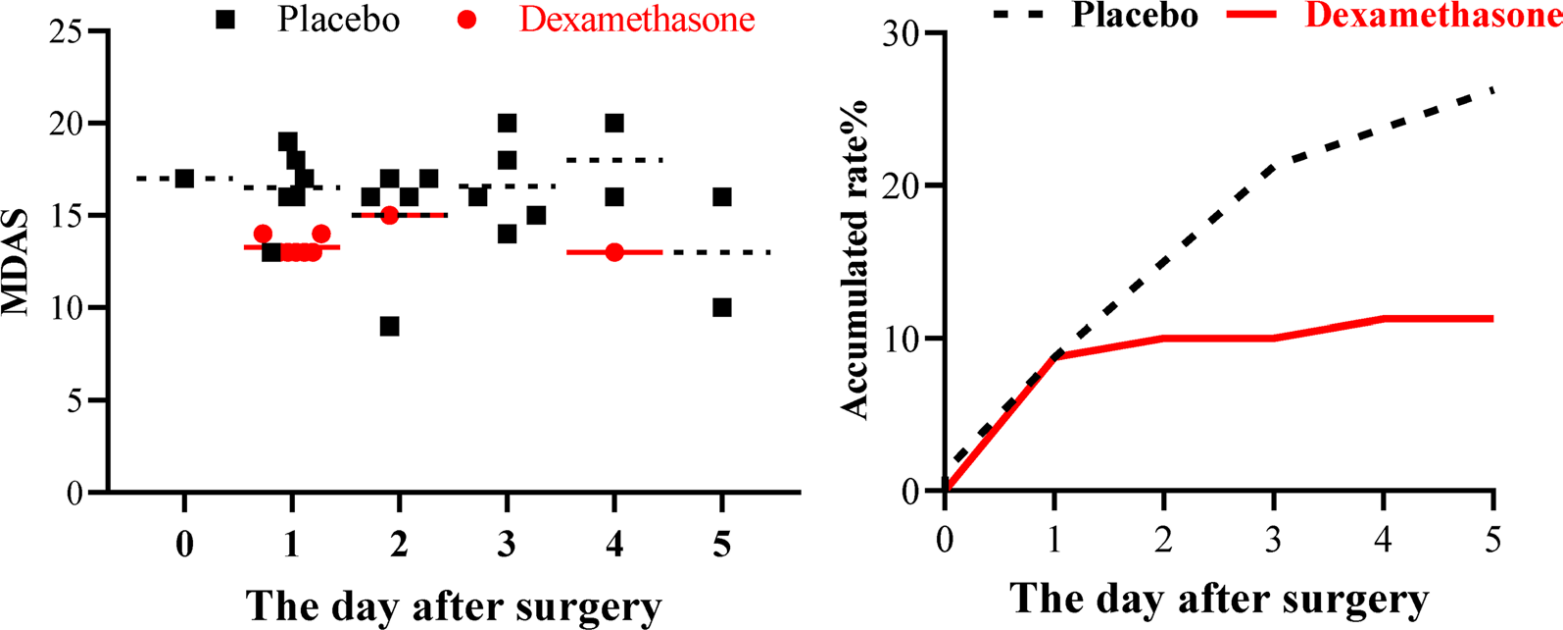
Comparison of incidence and severity of POD between two groups on the five days after surgery

The incidence and severity of POD in the first 5 days after surgery are presented in Table 4 and Fig. 2. The comparison of POD incidence in the first 5 days after surgery is shown in Table 4. No significant differences were observed in the incidence on each day in the first 5 days after surgery. Although there was a lower incidence of the POD in the first 3 days in the dexamethasone group than in the placebo group (8/80 10.0%, vs. 17/80, 21.3%), the difference did not reach significant (*P* = 0.05; RR = 0.88, 95% CI 0.76–1.00).

**Fig. 3:**
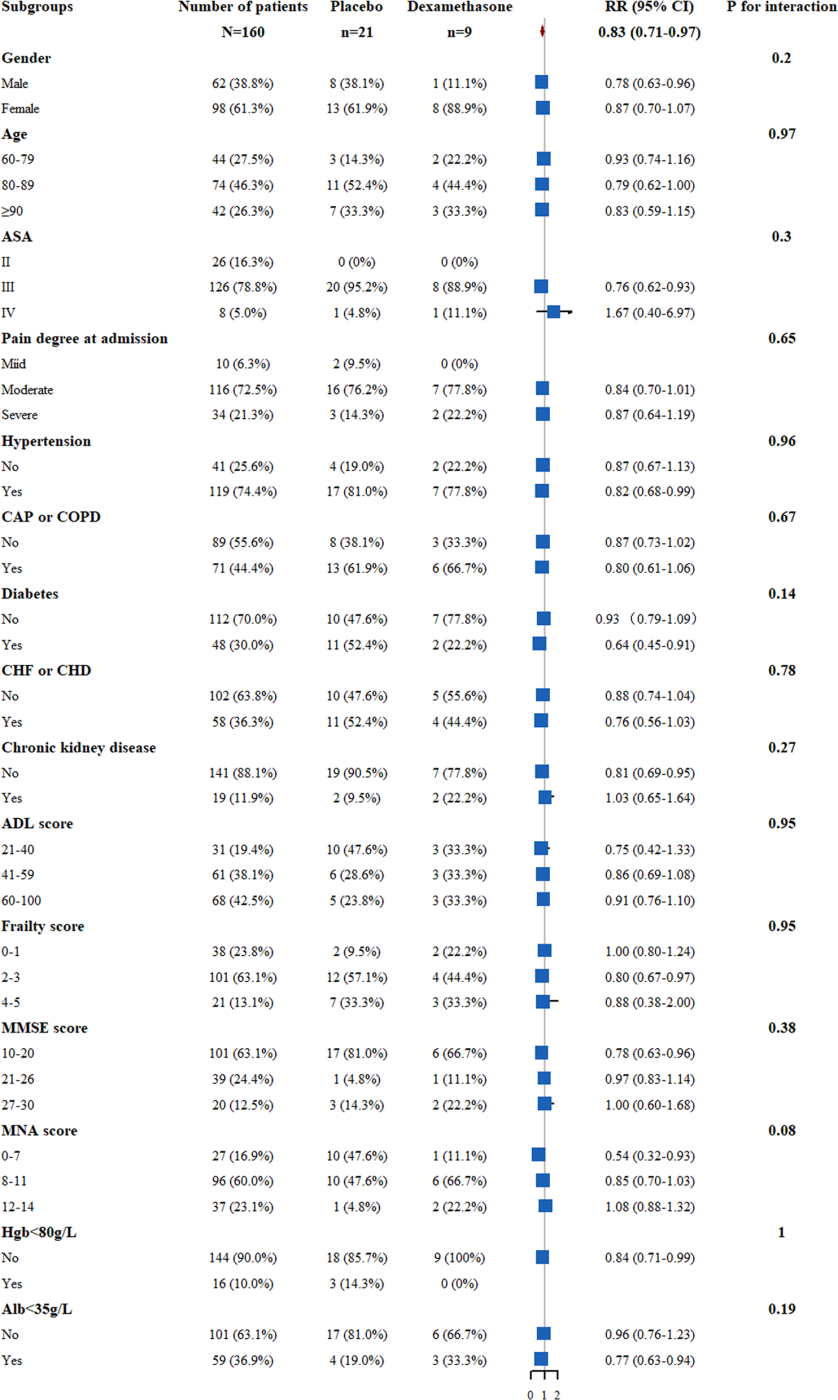
Relative risk for the POD in post hoc subgroups analysis. There were no significant interactions in all the 11 subgroups (*P* > 0.05 for all comparison)

Post hoc subgroup analysis was shown in Fig. 3. No significant interaction effect was observed in the two groups and all the 11 subgroups.

## Discussion

POD is a distributing problem in clinical practice, of which the pathogenesis, preventions, and treatments are still unclear [7]. Furthermore, POD is a complication associated with high prevalence and poor prognosis [26]. This single-blind, randomized control trial indicates that a single preoperative low-dose dexamethasone is beneficial in reducing both the incidence and the severity of POD in geriatric ITF after internal fixation surgery. The results provide theoretical basics for dexamethasone administration in optimizing ERAS clinical pathways of ITF.

Previous studies indicated that the incidence of POD in hip fractures varied from 14.3 to 25.0% [4–6]. Our study showed that the incidence of POD was 26.3% (21/80) in the placebo group, which is essentially in accordance with that reported in the literature [4–6]. The recognition and diagnosis of POD were great clinical concerns as the opinions divided into which tool is the best for evaluating POD [26–28]. In our study, Nu-DESC and MDAS were regarded as the screening measure of POD incidence and quantitative assessment of POD severity, respectively. Nu-DESC is the most useful method with high sensitivity for early diagnosis of POD, which is not required specific training to ensure optimum performance [27, 29]. As the severity of POD could not be assessed by Nu-DESC, MDAS was used as a definitive evaluation of both the incidence and severity of POD for its’ superiority in assessing delirium [28, 29]. The combination of Nu-DESC and MDAS for identifying POD is both efficient and precise. An MDAS score of 7.5 or more indicates delirium or is correlated with cognitive dysfunction after hip fracture [30]. In our study, the MDAS score of patients diagnosed with POD was at least 9 points (9–20), which might indicate a high accuracy in detecting and diagnosing POD.

Infections events and hyperglycemia are associated with the long-term use of high-dose steroids, but there is currently no clear evidence that a single low-dose dexamethasone given preoperatively increases these adverse events. A meta-analysis involving 51 studies confirmed that a single preoperative high-dose, short-acting corticosteroid did not increase the risk of postoperative complications after trauma surgery [31]. In this study, a higher pneumonia rate was found in the dexamethasone group, but the difference was not statistically significant. Prior studies have reported that low-dose dexamethasone used in arthroplasty surgery did not increase infection events [32, 33], yet whether high-dose dexamethasone would result in infection is still debatable and unclear [17, 34, 35]. A recent study concluded that a high dose of preoperative dexamethasone could potentially increase postoperative infection in long-term follow-up in hip fractures [17]. Besides, although no significant difference, higher hyperglycemia rates, and higher maximum glucose were found in the dexamethasone group in this study. Our findings are consistent with prior studies showing that blood glucose was raised after a single perioperative low dose of dexamethasone but there was no apparent increase in adverse effects [33, 35–37]. However, some studies have suggested that dexamethasone used in patients with diabetes mellitus should be prudent because of the uncertain effect. Thus, both the correlation between the dose and the infection and the effect of low-dose dexamethasone on patients with diabetes mellitus should be further investigated in future studies for their clinical significance.

The most important finding in this study is a single, preoperative, low-dose dexamethasone significantly reduced both the incidence and severity of POD in the first 5 days after surgery in geriatric ITF patients. The potential mechanism for this finding is dexamethasone reduces some POD-related inflammatory molecules like IL-1β by exerting anti-inflammatory effects [38, 39]. Besides, as the high concentration of endogenous cortisol has been demonstrated previously to be associated with POD [40], there is a possibility that preoperative dexamethasone could reduce endogenous cortisol levels by negative feedback of the hypothalamic‑pituitary‑adrenal axis [41]. But on the other hand, the between-group comparisons of daily POD incidence did not reach statistical significance. The reason for this observation may be partly attributed to the low incidence each day. Besides, the daily POD incidence in our study was the new-onset POD on that day, and the assessment of patients with POD was not consecutive and dynamic. We speculated that it would be lesser helpful for significant differences in daily incidence between the two groups.

There were some differences between our study and previous similar studies [16, 17]. Firstly, the observation duration of POD in this study was increased from 3 to 5 days after surgery to reduce the missed diagnosis of POD [29]. Our study found that the decrease in POD incidence was also observed in the first 3 days after surgery (10.0% vs. 21.3%), attributed to the half-life of dexamethasone being nearly 36–72 h [42]. However, Kluger’s study showed that high-dose dexamethasone could significantly reduce the POD severity but did not affect the POD incidence [17]. That could presumably explain the timing of dexamethasone administration. In Kluger’s study, dexamethasone was given as early as possible following hospital admission to reduce the neuro-inflammatory response to the initial trauma and surgery. However, that was probably too soon for the geriatric patients in such a high dose (20 mg), as both the comorbidities and rapid change of patient condition after fracture might prolong the timing of surgery. Secondly, our current study excluded patients with severe cognitive impairment (as assessed with MMSE) while Kluger’s study excluded the patients with cognitive impairment (as assessed with the 4AT test). However, cognitive impairment might result in a diverse effect on POD incidence as preoperative cognitive impairment is one of the foremost inducing factors for POD [4–6]. Then, the time from dexamethasone intervention to surgery varied considerably (0.3–46.8 h) in Kluger’s study, which might probably cause different effects of dexamethasone on initial fracture and subsequent surgery. Finally, the detection of delirium was performed twice a day (at 8 h intervals) in our study while once a day in Kluger’s study, which might reduce the risk of missed diagnosis of POD. Unlike some previous studies, the observation duration of POD in this study increased from 3 to 5 days after surgery to reduce the missed diagnoses in POD, as reported by the European Society of Anesthesiology evidence-based guidelines. Also, five patients with POD were observed in this study on the 4th and 5th day postoperatively, which led to statistical differences in the comparisons of POD accumulated incidence between the two groups in the first 4 or 5 days after surgery.

Although previous studies have reported that advanced age, gender, ASA grade, nutrition, comorbidities, preoperative cognitive disorder, preoperative anemia, pain degree, the timing of surgery, and surgery duration are the risk factors for POD [4–7, 43, 44], our result showed that there were no significant interactions between the two groups and in the subgroups (all *p* values for interaction were over 0.05) except for nutrition status. Limited by the sample size and uncertainty of emergency, the prior subgroup design was difficult to complete in a single center. Therefore, the post hoc subgroup analysis was performed to explore the effect of dexamethasone on POD in different subgroups. Our result showed no significant interactions between the dexamethasone and subgroups (all *p* values for interaction were over 0.05) except for nutrition status. Although no significant interaction effect (*P* = 0.08), lower POD incidence was observed in patients with malnutrition compared to the patients without malnutrition after dexamethasone intervention (RR = 0.54, 95 CI% 0.32–0.93). The reason is likely that the effect of physiological stress after fracture and surgery would be more pronounced in the patients without malnutrition, which is likely correlated with the poorly compensated function of the systemic organ. Also, malnutrition would induce glucocorticoid resistance and compromise the protective response to stress that is afforded by endogenous cortisol [45]. Furthermore, a significant fraction of cortisol activity receptors translocated to the nucleus, and the increased expression of inactive receptors was observed in malnutrition [45, 46], which might reduce the combination and activity of cortisol, but the mechanism that nutrition how influences the effect of corticosteroids on POD remains unknown. Certainly, this current study was done post hoc, and these results should be interpreted with caution as it is an exploratory analysis.

There were still several limitations in our study. First, the sample size was relatively small, and the power calculation was not precisely performed a priori, which is an inevitable drawback of this study. Although two different assessments (Nu-DESC and MDAS) and a relatively long observation duration for increasing the possibility of finding a POD were used to evaluate POD, these results should be interpreted as preliminary and hypothesis-driven for future studies. Second, the subjective bias of the investigator might be inevitable because of the single-blinded design. Third, patients with severe cognitive impairment who might have the highest risk of developing POD and suffer the greatest benefit from the intervention were not included in our study due to the protection of vulnerable groups. Fourth, anesthetic management without prespecified anesthetic protocols was left to the discretion of treating anesthesiologists, which might have a potential effect on the intervention. Fifth, prespecified stratified randomization was not performed at the recruitment, thereby further inferential conclusions about the impact of dexamethasone on subgroups could not be drawn. Finally, an increased probability of at least one false significant result might be inevitable due to the characteristic of secondary outcomes analysis. Therefore, the conclusion should necessarily be exploratory.

In conclusion, a single preoperative low-dose dexamethasone may reduce both the incidence and the severity of POD in geriatric ITF patients with internal fixation surgery.

## Supplementary Information

**Additional file 1**. The ERAS procedure, Diagnostic criteria of safety-related outcomes, and Postoperative adverse events during hospitalization.

## Data Availability

The dataset supporting the conclusions of this study is available upon request by contacting the corresponding author, but the primary data were not shared because other studies related these primary data were underway confidentially.

## Code availability

Software applications used in this study are listed as follows: IBM SPSS Statistic for Windows, Version 25.0. Armonk, NY: IBM Corp; R.app GUI 1.79 (8160 High Sierra build), S. Urbanek & H.-J. Bibiko, © R Foundation for Statistical Computing, 2021 RStudio 2022.02.3 Build 386 © 2009–2023 Posit Software, PBC.
