# Supplementary material for: A single preoperative low-dose dexamethasone may reduce the incidence and severity of postoperative delirium in the geriatric intertrochanteric fracture patients with internal fixation surgery: an exploratory analysis of a randomized, placebo-controlled trial
Source: J Orthop Surg Res. 2023 Jun 19;18:441. doi: 10.1186/s13018-023-03930-2 (PMC10280884; doi:10.1186/s13018-023-03930-2)
Supplement: Supplementary file 1 — Additional file 1. The ERAS procedure, Diagnostic criteria of safety-related outcomes, and Postoperative adverse events during hospitalization. [file 13018_2023_3930_MOESM1_ESM.docx]

1. **Standard ERAS procedure**

| **Preoperative management** | |
| --- | --- |
| Education program | Collect medical history to assess patient condition  Nutrition, psychological, nursing and surgery education  Advocate pulmonary exercises and timely assisted to turn over  Advocate function activity of no-injured limb |
| Auxiliary examination | Complete preoperative laboratory tests and imaging examinations within 24 hours after admission  Individualized specialty consultations or MDT 24 hours within admission |
| Nutrition management | High protein diet or use human serum albumin when necessary  Blood transfusion when Hgb < 70g/L or Hgb < 80g/L with anemia symptoms |
| Pain management | Routine use of NASID for example imrecoxib 200 mg Po Bid  Intravenous administration of NASID or opioid when necessary |
| DVT Prevention | Subcutaneous LMWH 0.3-0.4 ml Qd |
| Sleep management | Sedative hypnotic such as alprazolam 0.4 mg Qn |
| Gastrointestinal management | Routine use of oral PPI and water-swallowing test  A 6-hours fasting and 2-hours water fasting period before surgery |
| **Intraoperative management** | |
| Anesthesia | General anesthesia (laryngeal mask or endotracheal intubation) or spinal anesthesia done by experienced anesthesiologists |
| Surgery optimization | Close reduction or limited open reduction  Minimize the use number of C-arm as much as possible  Close skin with intracutaneous suture |
| Temperature management | Dynamically monitor operating room temperature  Reduce limb exposure during surgery  Keep both patient body and infusion liquid warming with attemperators |

| **Postoperative management** | |
| --- | --- |
| Pain management | Routine use of NASID for example imrecoxib 200 mg Po Bid  Use NASID or opioid alternatively according to pain degree |
| DVT Prevention | Subcutaneous LMWH 0.3-0.4ml Qd； |
| Respiratory management | Route nebulization for expectoration  Advocate deep breathing and blow balloon  Complete chest radiography within 48 hours after surgery |
| Functional exercise | Passive exercise of lower extremity with human help or CPM  Active exercise of lower extremity joints 48 h after surgery  Appropriate Ambulatory rehabilitation exercise |
|  | Timely turn over and clap back at 8 h intervals |
|  | Route swallowing function exercise |

1. **Diagnostic criteria of safety-related outcomes.**

**Pulmonary infections^1^**

| Major criterion | The presence of new and/or progressive pulmonary infiltrates on chest radiograph that occurs more than 48 hours after admission |
| --- | --- |
| Minor criteria | Fever ≥ 38.5°C or hypothermia <36°C |
|  | WBC ≥ 12*10^9/L or WBC ≤ 4*10^9/L |
|  | Purulent sputum |
|  | New onset or worsening cough |
|  | Dyspnea |

Major criterion and at least two minor criteria were required for a diagnosis.

**Wound infections^2^**

Wound infection included superficial and deep surgical site infection.

**Urinary infections^3^**

The criteria for urinary infections were: > 10 white blood cells/high-power field on microscopic examination, or isolation of > 105 organisms/ml urine, or > 104 organisms with symptoms.

**Sepsis** was defined as at least 2 of the following first 4 criteria and fifth criterion**^4^**

1. Axillary temperature >38.5°C or <36.5°C.

2. Heart rate >90 beats/minute. If patient had an atrial arrhythmia, record the ventricular rate. If patients have a known medical condition or are receiving treatment that would prevent tachycardia (for example, heart block or beta blockers), they must meet two of the remaining three SIRS criteria

3. Respiratory rate >20 breaths/minute or a PaCO2 <32 mmHg or mechanical ventilation for an acute process.

4. White blood cell (WBC) count of >12 x 10^9/L or <4 x 10^9L

5. Defined focus of infection is indicated by either an organism grown in blood or sterile

site, or an abscess or infected tissue (including pneumonia, peritonitis, urinary tract, vascular line infection, soft tissue, etc.)

**Hyperglycemia^5^**

The criteria for hyperglycemia were fasting blood glucose ≥7mmol or 2-hour postprandial blood glucose ≥11mmol/L or random blood glucose ≥11mmol/L.

1. **Postoperative adverse events during hospitalization between the two groups (values are presented as number (%))**

|  | **Placebo group (N=80)** | **Dexa group (N=80)** | ***P* value** |
| --- | --- | --- | --- |
| **Skin and issue disorders** | 3 | 1 |  |
| Ulcer | 3 (3.8) | 1 (1.2) | 0.613^Y^ |
| **Nervous system disorders** | 11 | 7 |  |
| Insomnia | 8 (10.0) | 7 (8.8) | 0.786^P^ |
| Delirium^a^ | 2 (2.5) | 1 (1.3) | 1^Y^ |
| Cerebrovascular event | 3 (3.8) | 0 (0.0) | 0.244^Y^ |
| **Blood system** | 37 | 38 |  |
| Anemia (blood transfusion) required) | 37 (46.2) | 38 (47.5) | 0.874^P^ |
| **Respiratory disorders** | 21 | 12 |  |
| Hypoxia | 8 (10.0) | 6 (7.5) | 0.576^P^ |
| Hypercapnia | 5 (6.2) | 2 (2.5) | 0.440^Y^ |
| Acute respiratory failure (type I) | 5 (6.2) | 3 (3.8) | 0.717^Y^ |
| Acute respiratory failure (type II) | 3 (3.8) | 1 (1.2) | 0.613^Y^ |
| **Cardiac disorders** | 26 | 24 |  |
| Bradycardia | 1 (1.2) | 1 (1.2) | 1^Y^ |
| Tachycardia | 14 (17.5) | 17 (21.2) | 0.548^P^ |
| Arterial fibrillation | 1 (1.2) | 1 (1.2) | 1^Y^ |
| Acute heart failure | 8 (10.0) | 5 (6.2) | 0.385^P^ |
| Acute myocardial infarction | 2 (2.5) | 0 (0.0) | 0.477^Y^ |
| **Gastrointestinal disorders** | 35 | 16 |  |
| Nausea and vomiting | 29 (36.2) | 12 (15.0) | 0.002^P^ |
| Abdominal pain | 3 (3.8) | 0 (0.0) | 0.244^Y^ |
| Positive occult blood test | 3 (3.8) | 4 (5.0) | 1^Y^ |
| **Renal and urinary disorders** | 10 | 9 |  |
| Urinary retention | 10 (12.5) | 9 (11.2) | 0.807^P^ |
| **Blood glucose disorders** | 3 (3.8) | 0 (0.0) |  |
| Hypoglycemia | 3 (3.8) | 0 (0.0) | 0.244^Y^ |
| **Vascular disorders** | 50 | 29 |  |
| Hypotension | 3 (3.8) | 4 (5.0) | 1^Y^ |
| Hypotension, need for Dopamine | 2 (2.5) | 0 (0.0) | 0.477^Y^ |
| Hypertension |  |  |  |
| Oral antihypertensive drug | 12 (15.0) | 9 (11.2) | 0.482^P^ |
| Sublingual nitroglycerin | 8 (10.0) | 6 (7.5) | 0.576^P^ |
| Intravenous drip nitroglycerin | 3 (3.8) | 2 (2.5) | 1^Y^ |
| Deep venous thrombosis | 3 (3.8) | 2 (2.5) | 1^Y^ |
| **Metabolism disorders** | 118 | 82 |  |
| Hypokalemia | 26 (32.5) | 13 (16.2) | 0.017^P^ |
| Hyperkalemia | 4 (5.0) | 1 (1.2) | 0.363^Y^ |
| Hyponatremia | 28 (35.0) | 23 (28.7) | 0.396^P^ |
| Hypernatremia | 6 (7.5) | 4 (5.0) | 0.514^P^ |
| Hypocalcemia | 54 (67.5) | 41 (51.2) | 0.036^P^ |

^a^ Delirium occurred later than the first five days after surgery

^p^ Pearson's chi-squared test, ^Y^ Yate’s continuity correction

**Reference**

1. Asehnoune K, Le Moal C, Lebuffe G, et al. Effect of dexamethasone on complications or all cause mortality after major non-cardiac surgery: multicentre, double blind, randomised controlled trial. *BMJ (Clinical research ed)*. Jun 2 2021;373:n1162. doi:10.1136/bmj.n1162

2. Ban KA, Minei JP, Laronga C, et al. American College of Surgeons and Surgical Infection Society: Surgical Site Infection Guidelines, 2016 Update. *Journal of the American College of Surgeons*. Jan 2017;224(1):59-74. doi:10.1016/j.jamcollsurg.2016.10.029

3. Zhang AQ, Zeng L, Gu W, et al. Clinical relevance of single nucleotide polymorphisms within the entire NLRP3 gene in patients with major blunt trauma. *Critical care (London, England)*. 2011;15(6):R280. doi:10.1186/cc10564

4. Levy MM, Fink MP, Marshall JC, et al; SCCM/ESICM/ACCP/ATS/SIS. 2001 SCCM/ESICM/ACCP/ATS/SIS International Sepsis Definitions Conference Crit Care Med. 2003; 31:1250-6

5. Alberti KG, Zimmet PZ. Definition, diagnosis and classification of diabetes mellitus and its complications. Part 1: diagnosis and classification of diabetes mellitus provisional report of a WHO consultation. *Diabetic medicine : a journal of the British Diabetic Association*. Jul 1998;15(7):539-53. doi:10.1002/(sici)1096-9136(199807)15:7<539::Aid-dia668>3.0.Co;2-s
